# CaMKK2: bridging the gap between Ca^2+^ signaling and energy-sensing

**DOI:** 10.1042/EBC20240011

**Published:** 2024-11-18

**Authors:** Luke M. McAloon, Abbey G. Muller, Kevin Nay, Eudora L. Lu, Benoit Smeuninx, Anthony R. Means, Mark A. Febbraio, John W. Scott

**Affiliations:** 1 Drug Discovery Biology, Monash Institute of Pharmaceutical Sciences, Parkville, Victoria 3052, Australia; 2 Medicinal Chemistry, Monash Institute of Pharmaceutical Sciences, Parkville, Victoria 3052, Australia; 3 Molecular and Cellular Biology, Baylor College of Medicine, Houston, TX 77030, U.S.A.; 4 St Vincent's Institute of Medical Research, Fitzroy, Victoria 3065, Australia

**Keywords:** AMPK, calcium signaling, calmodulin, CaMKK2, energy-sensing

## Abstract

Calcium (Ca^2+^) ions are ubiquitous and indispensable signaling messengers that regulate virtually every cell function. The unique ability of Ca^2+^ to regulate so many different processes yet cause stimulus specific changes in cell function requires sensing and decoding of Ca^2+^ signals. Ca^2+^-sensing proteins, such as calmodulin, decode Ca^2+^ signals by binding and modifying the function of a diverse range of effector proteins. These effectors include the Ca^2+^-calmodulin dependent protein kinase kinase-2 (CaMKK2) enzyme, which is the core component of a signaling cascade that plays a key role in important physiological and pathophysiological processes, including brain function and cancer. In addition to its role as a Ca^2+^ signal decoder, CaMKK2 also serves as an important junction point that connects Ca^2+^ signaling with energy metabolism. By activating the metabolic regulator AMP-activated protein kinase (AMPK), CaMKK2 integrates Ca^2+^ signals with cellular energy status, enabling the synchronization of cellular activities regulated by Ca^2+^ with energy availability. Here, we review the structure, regulation, and function of CaMKK2 and discuss its potential as a treatment target for neurological disorders, metabolic disease, and cancer.

## Introduction

Calcium ions (Ca^2+^) serve as universal carriers of biological information that direct a vast array of functions crucial for regulating cell proliferation, growth, metabolism, and immunity [1]. Ca^2+^ signaling is critical for survival, enabling cells to quickly sense and respond to changing environmental conditions and pathogens [2]. A key aspect of Ca^2+^ signaling involves translating information encoded in Ca^2+^ signals into specific cellular responses. The ubiquitous Ca^2+^-sensing protein, calmodulin, plays an essential role in this process [3]. It decodes Ca^2+^ signals by binding to and altering the activity of a broad range of effector proteins in response to increased intracellular Ca^2+^ levels.

**Figure 1:**
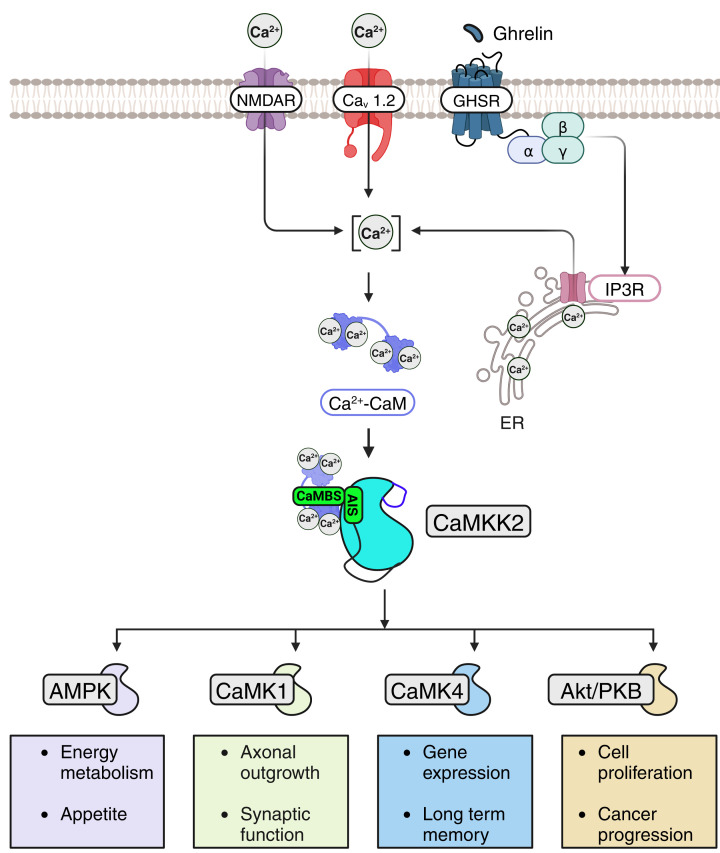
CaMKK2 signaling pathway CaMKK2 is activated by voltage and ligand-gated Ca^2+^ channels (Ca_V_1.2 and NMDA receptor) as well as hormone receptors (GSHR) that increase intracellular Ca^2+^ and cause accumulation of the Ca^2+^-calmodulin complex. CaMKK2 decodes Ca^2+^ signals generated by these cell surface activators by triggering the downstream activation of the CaMK1, CaMK4, AMPK, and Akt/PKB signaling pathways. Through these downstream pathways, CaMKK2 regulates a variety of cellular processes that support brain function and whole-body energy homeostasis.

The enzyme Ca^2+^-calmodulin-dependent protein kinase kinase-2 (CaMKK2) is one of several known protein kinase effectors of the Ca^2+^-calmodulin complex, and is activated in response to membrane depolarisation, as well as neurotransmitters and hormones, that increase intracellular Ca^2+^ (Figure 1) [4]. Cell surface activators of CaMKK2 include the voltage-gated Ca_v_1.2 channel, the NMDA receptor, and the ghrelin receptor [5–7]. CaMKK2 converts Ca^2+^-signals generated by these receptors into cellular responses by activating four downstream signaling pathways, which are regulated by the Ca^2+^-calmodulin-dependent protein kinases 1 and 4 (CaMK1 and CaMK4), AMP-activated protein kinase (AMPK), and Akt/protein kinase B (PKB) [8–14]. CaMKK2 activates each substrate by phosphorylating a key threonine residue within the regulatory activation loops of their kinase domains. Through these substrates, CaMKK2 regulates a variety of cellular activities essential for maintaining brain function and whole-body energy homeostasis [15].

The CaMKK2-AMPK axis serves as an important signaling node that integrates Ca^2+^ signals with cellular energy status [10,11,16]. AMPK is a heterotrimeric complex composed of an α catalytic subunit, and regulatory β and γ subunits of which there are multiple isoforms [17]. It functions as an energy-sensor and is activated by metabolic stresses that increase the cellular AMP-to-ATP ratio, which typically occurs during conditions of high-energy demand or low nutrient availability. Long-chain fatty acid metabolites such as palmitoyl-CoA can also activate AMPK, but this additional layer of allosteric activation is restricted to AMPK complexes containing the β1 subunit isoform [18]. Although CaMKK2 can activate AMPK independently of changes in cellular energy status, activation is enhanced when AMP levels are elevated [19–21].

In this review, we provide an update on the current state of the CaMKK2 field and discuss the role of CaMKK2 as a signaling hub for multiple signal transduction pathways. We also review the role of the CaMKK2-AMPK pathway in the regulation of whole-body energy metabolism and brain function, and comment on emerging evidence that suggests paradoxical roles for CaMKK2 and AMPK in fatty liver disease and prostate cancer.

## CaMKK2 structure and allosteric regulation

**Figure 2:**
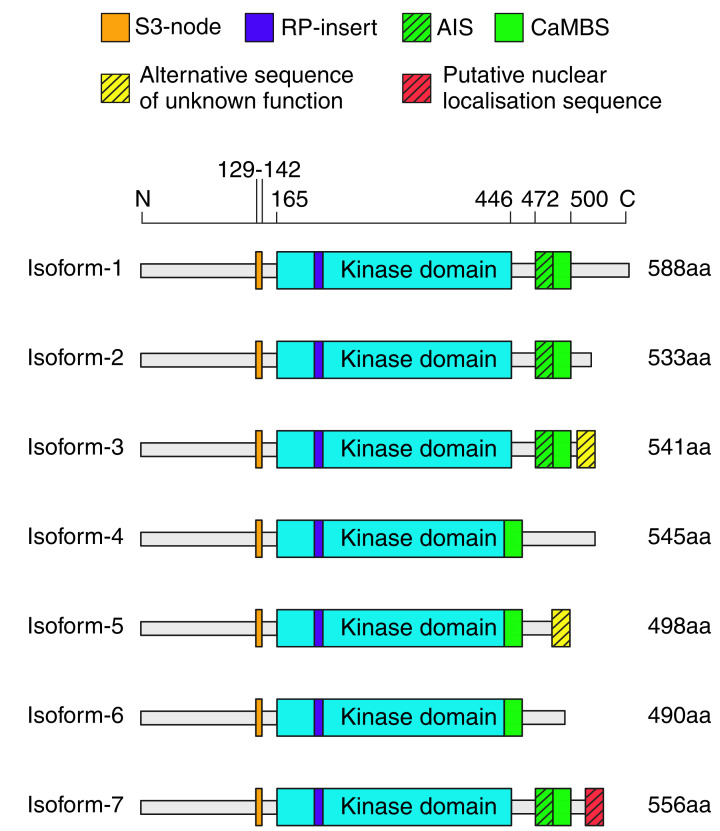
Domain structure of CaMKK2 protein isoforms The domain structure of the seven known protein isoforms of human CaMKK2 displaying the location of the kinase domain (cyan), RP-insert (blue), the partially overlapping autoinhibitory (AIS; green and black stripes) and calmodulin-binding sequences (CaMBS; green), as well as the regulatory S3-node (orange). The N-terminal regions of each CaMKK2 isoform are highly-conserved but they differ in their C-terminal sequences. Of note, isoform-3 has an alternative C-terminal sequence of unknown function (yellow and black stripes). Isoform-7 also has an alternative C-terminal sequence (red and black stripes) that is unique from the other isoforms that contains a putative nuclear localisation sequence (NLS).

CaMKK2 has a modular architecture composed of a central kinase domain and a regulatory region containing overlapping autoinhibitory (AIS) and calmodulin-binding sequences (CaMBS), which are surrounded by N- and C-terminal sequences of unknown function (Figure 2). It also contains an arginine-proline (RP) rich sequence insert within the small N-lobe of the kinase domain that is unique to CaMKK2 and its paralogue, CaMKK1. The RP-insert has been reported to play an important role in enabling CaMKK2 to target and activate CaMK1, CaMK4, and AMPK [22].

CaMKK2 protein has been detected in a variety of human tissues including brain, liver, kidney, bone marrow, gastrointestinal tract, and both male and female reproductive tissues [23]. Alternative RNA splicing of the gene that encodes human CaMKK2 results in seven known protein isoforms, which vary only in their C-terminal sequences (Figure 2). At present, little is known about the regulation of these different isoforms or if they have distinct functions. There is some confusion regarding the naming of these isoforms, as Uniprot and the National Centre for Biotechnology Information (NCBI) use different numbering systems. For consistency and clarity in future research, we recommend using the Uniprot naming convention when referring to the various CaMKK2 isoforms [24]. Isoform-1 (Uniprot: Q96RR4) is the most abundant and canonical isoform of CaMKK2 expressed in humans, and is highly expressed in the brain [25]. Isoform-2 (Q96RR4-2) displays nominal expression in the brain and is largely undetectable in peripheral tissues. It is identical in sequence to isoform-1, except that it prematurely terminates at residue 533. Despite lacking a significant proportion of the C-terminal sequence, isoform-2 displays similar kinase activity and Ca^2+^-calmodulin activation to isoform-1 [26]. Isoform-3 (Q96RR4-3) contains an alternative C-terminal sequence and is the predominant isoform in prostate tissue (both with and without cancer) [27]. Isoforms-4 (Q96RR4-4), -5 (Q96RR4-5) and -6 (Q96RR4-6) display modest levels of expression in the brain compared with isoform-1, and lack a small section of sequence at the end of their kinase domains, which may impact kinase activity [25]. Isoform-7 (Q96RR4-7) features a unique alternative C-terminal sequence, distinct from the alternative sequence in isoform-3, and shares a similar expression pattern with isoform-1 [25]. The unique C-terminal sequence of isoform-7 contains a putative nuclear localisation sequence (NLS), but it remains unknown whether this isoform resides in the nucleus. Interestingly, a recent whole exome sequencing study of a cohort of early-onset and late-onset obesity patients identified a novel variant, Gly539ArgfsTer3, of isoform-7 [28]. This variant is proposed as a potential monogenic driver of obesity, which suggests an important role for isoform-7 of CaMKK2 in the regulation of whole-body energy metabolism.

Ca^2+^-calmodulin was originally thought to activate CaMKK2 by preventing the autoinhibitory sequence (which overlaps with the calmodulin-binding sequence) from occluding the substrate-binding pocket in the active site of the kinase domain, similar to the activating mechanism observed in other members of the CaMK family [13,29,30]. However, the crystal structure of the kinase domain of CaMKK2 revealed that its C-lobe lacks an αD helix, a key structural element involved in interactions with autoinhibitory sequences in other protein kinases, such as CaMK1 and CaMK2 [31]. These findings indicate that interactions involving the kinase domain, autoinhibitory sequence, and calmodulin-binding sequence of CaMKK2, and consequently its Ca^2+^-calmodulin activation mechanism, may differ significantly from other calmodulin dependent kinases. Supporting this idea, recent structural studies using chemical crosslinking and hydrogen-deuterium exchange mass spectrometry found that the autoinhibitory sequence of CaMKK2 does not block the substrate-binding pocket [32]. Instead, it maintains CaMKK2 in an autoinhibited state by interacting with surfaces on the opposite side of the kinase domain, away from the active site. This study also revealed that Ca^2+^-calmodulin interacts not only with the calmodulin-binding sequence but also with the small N-lobe, RP-insert, and activation loop of CaMKK2. These interactions are thought to be critical in assembling the catalytic spine, a key feature of an activated protein kinase, into an optimal conformation that enhances CaMKK2 activity [33,34].

## CaMKK2 is a signaling hub for multiple signal transduction pathways

**Figure 3:**
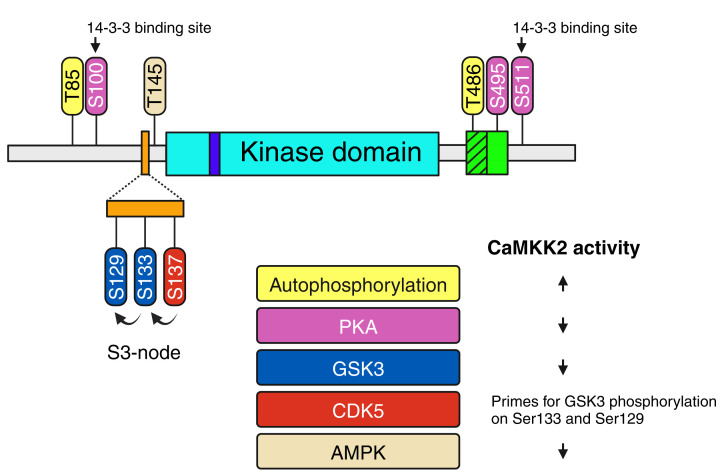
CaMKK2 is a signaling hub for multiple signal transduction pathways The domain structure of human CaMKK2 showing the positions of the regulatory phosphorylation sites. Autophosphorylation of Thr85 and Thr486 enhance CaMKK2 activity. In the S3-node, phosphorylation of Ser137 by CDK5 primes CaMKK2 for subsequent phosphorylation on Ser133 and Ser129 by GSK3, which decreases CaMKK2 activity. Phosphorylation of Ser100, Ser495 and Ser511 by PKA blocks CaMKK2 activation by Ca^2+^-calmodulin, and promotes the binding of 14-3-3 adaptor proteins, which keep CaMKK2 in an inactive state.

In addition to allosteric regulation by Ca^2+^-calmodulin, CaMKK2 is also regulated via multisite phosphorylation. This multi-level signal integration enables CaMKK2 to synchronize its activity with other major signaling pathways (Figure 3). Out of the twenty-two identified phosphorylation sites on CaMKK2, nine are known to have regulatory functions [35]. Among these, two are autophosphorylation sites: Thr85, located in the unstructured N-terminal sequence, and Thr486, located in the autoinhibitory sequence [36,37]. Currently, there is no evidence indicating whether the different protein isoforms of CaMKK2 undergo differential phosphorylation.

In human CaMKK2, binding of Ca^2+^-calmodulin induces autophosphorylation of Thr85 [36]. This creates a molecular memory that enables CaMKK2 to remain in its activated state beyond the initial transient Ca^2+^ signal. Thr85 autophosphorylation is important for optimal brain function, as two independent candidate gene association studies have shown a statistically significant link between bipolar and anxiety disorders, and a missense polymorphism that results in a Thr85Ser substitution at this site [38,39]. Unlike wild-type CaMKK2, the Thr85Ser variant undergoes autophosphorylation upon Ca^2+^-calmodulin binding but fails to sustain the activated state and instead causes a temporary loss of activity [36].

CaMKK2 displays significant basal activity in the absence of Ca^2+^-calmodulin whereas its paralogue, CaMKK1, is entirely dependent on Ca^2+^-calmodulin for kinase activity [40]. The higher basal activity of CaMKK2 is partly due to autophosphorylation of Thr486, which partially disrupts its autoinhibitory mechanism [37]. This process occurs independently of Ca^2+^-calmodulin and has been postulated to be required for CaMKK2-mediated activation of AMPK. This is because, except for the difference in basal activity, both CaMKK1 and CaMKK2 have almost identical biochemical properties and substrate specificity *in vitro*, yet only CaMKK2 activates AMPK* in vivo* [10,16].

The activation of CaMKK2 by Ca^2+^-calmodulin is fine-tuned via inhibitory crosstalk with the PKA signaling pathway. PKA phosphorylates Ser495 within the calmodulin-binding sequence of CaMKK2, which blocks Ca^2+^-calmodulin binding and prevents activation [41]. Additionally, PKA also phosphorylates Ser100 and Ser511, which facilitates the recruitment of 14-3-3 adaptor proteins that hold CaMKK2 in an inactive state by preventing the dephosphorylation of phospho-Ser495. Although death-associated protein kinase-1 (DAPK1) can also phosphorylate Ser511, this alone is insufficient for CaMKK2 to bind 14-3-3 proteins [41,42]. Nevertheless, Ser511 phosphorylation has been shown to inhibit Ca^2+^-calmodulin dependent autophosphorylation [42].

CaMKK2 activity is also modulated by hierarchical phosphorylation of three tandem serine residues (Ser129, Ser133 and Ser137) in the S3-node switch, which is a regulatory feature located in the N-terminal sequence of CaMKK2 [40,43]. The S3-node modulates CaMKK2 activity by regulating the interaction between the autoinhibitory sequence and kinase domain [40]. Phosphorylation of Ser137 by cyclin dependent kinase-5 (CDK5) primes CaMKK2 for subsequent phosphorylation on Ser133 and Ser129 by glycogen synthase kinase-3 (GSK3), which leads to a reduction in CaMKK2 basal activity. The CDK5 priming event is crucial and serves as a gatekeeping mechanism that enables GSK3 to suppress CaMKK2 activity [43]. Therefore, the S3-node acts as a two-input logic gate, inhibiting CaMKK2 activity only when both the CDK5 and GSK3 signaling pathways are activated.

Feedback regulation loops are a fundamental feature of signal transduction networks [44]. These loops enable signals to be amplified or dampened, and play a major role in ensuring signal fidelity. CaMKK2 is subject to negative feedback inhibition by AMPK through the phosphorylation of Thr145, situated between the S3-node and kinase domain of CaMKK2 [45]. Thr145 phosphorylation decreases the basal activity of CaMKK2 without impacting its maximal activation by Ca^2+^-calmodulin. As a result, Thr145 phosphorylation makes CaMKK2 become more dependent on Ca^2+^-calmodulin for its kinase activity.

In summary, the combination of multisite phosphorylation and allosteric regulation by Ca^2+^-calmodulin ensures precise control over CaMKK2 activation, allowing fine-tuning of the magnitude and duration of signal transmission.

## The CaMKK2-AMPK signaling axis regulates energy metabolism and brain function

The CaMKK2-AMPK signaling axis forms a crucial junction point that integrates Ca^2+^ signaling with cellular energy status, and plays a significant role in the regulation of whole-body energy metabolism and brain function [5,6,46].

CaMKK2 is highly expressed in the arcuate nucleus of the hypothalamus and is critical for regulating appetite [5]. During fasting, the appetite-stimulating hormone ghrelin is released from the stomach and binds to its cognate Gq-coupled growth hormone secretagogue receptor (GSHR) on neuropeptide-Y (NPY) neurons in the arcuate nucleus [47]. Activation of the ghrelin receptor increases intracellular Ca^2+^, leading to activation of the CaMKK2-AMPK pathway, which is necessary for promoting the expression of NPY and agouti-related protein (AgRP), both potent stimulators of appetite [47–50]. Mice lacking CaMKK2 do not respond to ghrelin, have reduced hypothalamic AMPK activity and NPY/AgRP expression, and consequently consume less food compared to pair-fed wild-type mice [5]. These findings also partially explain why CaMKK2 null mice are protected from high-fat diet-induced weight gain, glucose intolerance, and insulin resistance. In addition to regulating appetite, CaMKK2 also controls whole-body adiposity via regulation of adipogenesis. Activation of the CaMKK2-AMPK pathway in pre-adipocytes promotes the expression of Pref-1, which inhibits adipogenesis by preventing the differentiation of pre-adipocytes into mature adipocytes [51]. Loss of either CaMKK2 or AMPK activity enhances adipogenesis by promoting differentiation but does not affect cell proliferation.

An emerging body of data suggest that aberrant activation of the CaMKK2-AMPK pathway may be involved in the pathogenesis of neurodegenerative diseases such as Alzheimer’s disease, the most common form of dementia [52]. Amyloid plaques caused by the accumulation of amyloid-β 1-42 (Aβ42) peptides in the brain are a major hallmark of Alzheimer’s disease [53,54]. In the early stages of the disease, soluble oligomers of Aβ42 peptides cause the loss of excitatory synapses in cortical and hippocampal neurons, prior to the formation of insoluble amyloid plaques [55]. Mitochondrial dysfunction is also evident during the early phases of disease progression. Increased AMPK activity has been observed in the brain of patients with Alzheimer’s disease, and several studies have reported that Aβ42 oligomers can activate AMPK by a CaMKK2-dependent mechanism [56–58]. In hippocampal neurons, activation of the CaMKK2-AMPK pathway by Aβ42 oligomers has been shown to result in synaptic and dendritic spine loss, and increased phosphorylation of the microtubule-associated protein, Tau [6]. Hyperphosphorylation of Tau causes the formation of neurofibrillary tangles, another major hallmark of Alzheimer’s disease [59]. Significantly, genetic or pharmacological inhibition of CaMKK2 protected hippocampal neurons from the synaptotoxic effects of Aβ42 both *in vitro*, and in the human APP^SWD/IND^ transgenic mouse model of Alzheimer’s disease [6]. A follow-up study recently reported that Aβ42-mediated overactivation of the CaMKK2-AMPK pathway induces synaptic loss by promoting mitochondrial fission and mitophagy, which leads to a loss of mitochondrial biomass in neurons [60]. Significantly, these Aβ42-induced impairments in mitochondrial function were completely blocked by pharmacological inhibition of CaMKK2, which suggests that CaMKK2 inhibition may have therapeutic potential for treating Alzheimer’s disease.

## Paradoxical roles for CaMKK2 and AMPK in liver function

Paradoxical signaling is a common phenomenon in cell signaling whereby components of the same signaling pathway can induce opposite cell functions [61,62]. Accumulating evidence indicates that, under some conditions, activation of CaMKK2 and AMPK can have opposing physiological effects. For example, liver-specific deletion of CaMKK2 in mice protects against high-fat diet-induced glucose intolerance and insulin resistance, whereas genetic liver-specific AMPK activation has the same effect [63,64]. Furthermore, pharmacological inhibition of CaMKK2 with STO-609 has been reported to reverse key features of hepatic steatosis in two mouse models of fatty liver disease [65]. In contrast, two separate studies have demonstrated that activating AMPK with the small-molecule activator A769662 reverses hepatic steatosis in mouse models of fatty liver disease [66,67]. Although STO-609 has the same beneficial effects in liver as genetic deletion of CaMKK2, it’s important to note that STO-609 is not selective for CaMKK2 and can also inhibit AMPK [68]. Nevertheless, these findings demonstrate that the relationship between CaMKK2 activity and AMPK activation is not always linear. The exact mechanisms remain unclear, but it hints that CaMKK2 might be regulated by unidentified upstream signals that enable it to differentially engage distinct downstream pathways, perhaps in a manner similar to the biased signaling phenomenon observed in G-protein coupled receptors [69]. Additionally, since AMPK is also activated by liver kinase B1 (LKB1), CaMKK2 might play a less significant role in regulating AMPK in tissues like the liver, where LKB1 is highly expressed and considered to be the major upstream activator of AMPK [70,71].

## Paradoxical roles for CaMKK2 and AMPK in prostate cancer

CaMKK2 is a major target gene of the androgen receptor and plays a key role in driving prostate cancer progression [27,72,73]. Increased expression of CaMKK2 correlates with greater prostate cancer aggressiveness and poorer prognosis, and is associated with reduced patient survival [72,74]. Inactivation or loss of the phosphatase and tensin homolog (Pten) tumour suppressor also drives prostate cancer progression and is linked to more aggressive disease and shorter survival time [75]. Pten knockout mice (Pten KO) are widely used as a preclinical model as they accurately replicate the different stages of human prostate cancer [76]. Both CaMKK2 mRNA and protein expression have been reported to be up-regulated in Pten KO mice [77]. Notably, the same study showed that either pharmacological inhibition or genetic deletion of CaMKK2 provided protection against prostate cancer development. On the other hand, genetic deletion of the β1 subunit of AMPK in the Pten KO mice had the opposite effect, and promoted earlier onset of adenocarcinoma development [77]. These finding suggest, at least in the Pten mouse model, that CaMKK2 and AMPK have opposite effects. Indeed, proteomic analysis of prostate tissue from Pten KO mice lacking CaMKK2 revealed a significant reduction in the abundance of lipogenic proteins such as acetyl-CoA carboxylase-1 and fatty acid synthase [77]. This is significant, as increased de novo lipogenesis is a key driver prostate cancer. Consistent with these findings, lipogenesis was found to be reduced in prostate cancer cells lacking CaMKK2 compared with wild-type cells, whereas activation of AMPK also reduced lipogenesis in both wild-type and CaMKK2 knockout cells [77]. A separate study reported that activation of AMPK protects against prostate cancer by inducing a catabolic cellular state that results in reduced lipogenesis, increased fatty acid oxidation, and decreased cell proliferation [78]. Taken together, these findings demonstrate that CaMKK2 plays a key role in promoting lipogenesis in prostate cancer independently of AMPK. Although these studies suggest that AMPK activation protects against prostate cancer progression, others have yielded conflicting results. For example, research using patient tumour samples and a xenograft mouse model of castration-resistant prostate cancer found that disease progression was linked to increased CaMKK2-AMPK signaling [79]. Another study observed a similar increase in AMPK activation in patient-derived prostate cancer samples and showed that genetically deleting the α catalytic subunits of AMPK inhibited cell proliferation [80]. These contradictory findings might be due to a context-dependent role of AMPK in prostate cancer, therefore further investigation is needed to fully understand its function.

Overall, the physiological function of paradoxical signaling in the CaMKK2-AMPK pathway remains unclear. However, in other areas of biology such as the immune system, paradoxical signaling has been suggested to enable cells to dynamically respond to environmental fluctuations in order to maintain homeostasis [61,62].

## Concluding remarks

Despite significant advances in understanding the structure, regulation, and function of CaMKK2 since its discovery over three decades ago, it is still considered an understudied ‘dark’ kinase that is often eclipsed by its more extensively studied relatives in the CaMK family. Nevertheless, CaMKK2 has emerged as a pivotal decoder of Ca^2+^-signals in multiple tissues, and a key regulator of brain function and energy metabolism, with pathophysiological relevance for neurological disorders, metabolic diseases, and cancer. Given the compelling data implicating CaMKK2 as a major driver of prostate cancer, it is now considered a highly attractive therapeutic target [81]. Androgen deprivation therapy is the current standard of care for prostate cancer, but causes dyslipidaemia, insulin resistance, and sarcopenic obesity, thus mimicking metabolic syndrome [82]. It also reduces bone mass, increasing the risk of fractures [83]. However, CaMKK2 inhibition may offer a significant improvement over androgen deprivation therapy as it not only prevents prostate cancer progression but also protects against metabolic syndrome, improves insulin sensitivity, and increases bone mass [5,64,84]. Recent progress in developing highly selective and potent CaMKK2 inhibitors and ligand-directed degraders [85–90] has advanced the field a step closer to realizing the therapeutic potential of targeting CaMKK2 for treating prostate cancer and other diseases associated with aberrant CaMKK2 activation, including Alzheimer’s disease and liver steatosis.

## Summary

- CaMKK2 is a signaling hub for Ca^2+^ and kinase signal transduction pathways.
- CaMKK2 is a key regulator of energy metabolism and brain function.
- Paradoxical roles exist for CaMKK2 and AMPK in liver function and prostate cancer.
- CaMKK2 is a promising treatment target for prostate cancer.

## Open Access

Open access for this article was enabled by the participation of Monash University in an all-inclusive *Read & Publish* agreement with Portland Press and the Biochemical Society under a transformative agreement with CAUL.

## Abbreviations

AgRP: agouti-related protein
AIS: autoinhibitory sequence
AMP: adenosine-5′-monophosphate
AMPK: AMP-activated protein kinase
ATP: adenosine-5′-triphosphate
Aβ42: amyloid-β 1-42 peptide
CaMBS: calmodulin binding sequence
CaMK: Ca^2+^-calmodulin dependent protein kinase
CaMK1: Ca^2+^-calmodulin dependent protein kinase-1
CaMK4: Ca^2+^-calmodulin dependent protein kinase-4
CaMKK2: Ca^2+^-calmodulin dependent protein kinase kinase-2
CDK5: cyclin dependent kinase-5
CoA: coenzyme-A
DAPK1: death-associated protein kinase-1
GSHR: growth hormone secretagogue receptor
LKB1: liver kinase B1
NLS: nuclear localisation sequence
NMDA: N-methyl-D-aspartic acid
NPY: neuropeptide-Y
PKA: protein kinase A
PKB: protein kinase B
Pref-1: preadipocyte factor-1
Pten: phosphatase and tensin homologue
RP-insert: arginine-proline insert
